# Changing Functional Signatures of Microglia along the Axis of Brain Aging

**DOI:** 10.3390/ijms22031091

**Published:** 2021-01-22

**Authors:** Bianca Brawek, Maryna Skok, Olga Garaschuk

**Affiliations:** 1 Institute of Physiology, Department of Neurophysiology, Eberhard Karls University of Tübingen, 72076 Tübingen, Germany; bianca.brawek@uni-tuebingen.de; 2 Department of Molecular Immunology, Palladin Institute of Biochemistry NAS of Ukraine, 9, Leontovycha str., 01054 Kyiv, Ukraine; skok@biochem.kiev.ua

**Keywords:** microglia, calcium signaling, brain aging, discoordination of microglial processes, sex-specific differences, caloric restriction, acetylcholine receptors of α7 subtype, senescence, middle-age, in vivo calcium imaging

## Abstract

Microglia, the innate immune cells of the brain, are commonly perceived as resident macrophages of the central nervous system (CNS). This definition, however, requires further specification, as under healthy homeostatic conditions, neither morphological nor functional properties of microglia mirror those of classical macrophages. Indeed, microglia adapt exceptionally well to their microenvironment, becoming a legitimate member of the cellular brain architecture. The ramified or surveillant microglia in the young adult brain are characterized by specific morphology (small cell body and long, thin motile processes) and physiology (a unique pattern of Ca^2+^ signaling, responsiveness to various neurotransmitters and hormones, in addition to classic “immune” stimuli). Their numerous physiological functions far exceed and complement their immune capabilities. As the brain ages, the respective changes in the microglial microenvironment impact the functional properties of microglia, triggering further rounds of adaptation. In this review, we discuss the recent data showing how functional properties of microglia adapt to age-related changes in brain parenchyma in a sex-specific manner, with a specific focus on early changes occurring at middle age as well as some strategies counteracting the aging of microglia.

## 1. Introduction

The process of normal aging is accompanied by alterations in brain architecture. After the age of 50, brain weight decreases 2–3% per decade. As a consequence, brain weight in individuals of 80 years and older is typically 10% lower compared to that of young adults [1,2]. Although aging generally is not considered as a disease, it is a major risk factor for cognitive decline and various neurodegenerative diseases like Alzheimer’s disease (AD) or Parkinson’s disease (PD). Apart from brain atrophy, the senescent brain is considered to reside in a state of low-grade inflammation, a condition known as “inflammaging” [3]. This inflammatory state is likely caused by the ongoing stimulation of the immune system by internal or external factors, for example, long-lasting exposure to viral infection, presence of displaced or misfolded proteins of endogenous origin, cellular debris, etc. [4]. In addition, the brain is especially prone to accumulation of reactive oxygen species and oxidative stress because of its high energy demand and metabolic rate [2,5,6,7].

The aging-associated change in the inflammatory state of the brain is sensed by microglia, its major immune cells. Not surprisingly, these cells undergo the most prominent changes during the aging process. Microglia represent the first line of brain defense against endogenous and exogenous danger signals. They originate from erythromyeloid progenitors in the yolk sac, invade the brain during embryogenesis [8], and form a long-lived, autonomous, self-renewing population of cells [9]. Microglial cells exhibit a unique gene expression profile, which differentiates them from other CNS cells as well as other resident tissue macrophages. This unique identity is defined by their ontogeny as well as the influence of their specific environment in the CNS [10]. To fulfill their function as immune cells, microglia are equipped with a large repertoire of surface receptors enabling them to sense alterations in their environment [11]; e.g., expression of pattern recognition receptors enables them to detect danger-associated molecular patterns (DAMPs), like ATP or heat shock proteins, or pathogen-associated molecular patterns (PAMPs), like LPS [12,13,14].

As any disturbance of the brain environment profoundly affects the morphology and function of microglia, the morphological and functional phenotype of these cells changes along with the aging process. In the last years, it has become increasingly clear that the microglial population, present under specific conditions, is not homogeneous, but rather characterized by the presence of different phenotypes with unique properties [15,16,17]. Consistently, in the aging brain, senescent and dysfunctional as well as hyper-responsive and primed microglial phenotypes have been described.

Interestingly, the occurrence of the different microglial phenotypes seems to be sex-specific, with aging differently affecting the properties of microglia in males and females. In this context, it is important to note that gender is a predictor of susceptibility to several age-associated brain disorders. AD, for instance, has a higher (1.6–3:1) prevalence in women compared to men, whereas PD has a higher (3.5:1) prevalence in men compared to women [18]. Moreover, it seems that developmental disorders emerging early in life, e.g., schizophrenia or autism, are more common in males [19].

In this review, we look at brain aging from the microglia’s perspective, delineating different sex-specific microglial phenotypes, described in the course of the normal aging process, with a special focus on their morphology and surveillance as well as their Ca^2+^ signaling under in vivo settings. Specifically, we describe microglial phenotypes in young adult (3–6-month-old), middle-aged (10–14-month-old), and aged (16–24-month-old) mice. These age ranges correspond to the age of 20–30, 38–47, and 56–69 years in humans [20].

## 2. Functional Signature of Young Adult Microglia

### 2.1. Morphology and Surveillance

Microglia account for approximately 10% of all cells in the brain [21]. Although the microglial density remains largely constant throughout life, a certain degree of renewal and rearrangement has been reported [22,23]. Microglia in the young adult brain have a typical morphology, characterized by small somata and long, ramified processes. Microglial processes define a territorial domain of a single microglial cell, covering an area with a diameter of approximately 50 µm in the mouse as well as in the human brain [24]. Under physiological conditions, there is generally little overlap between territorial domains of microglia. Moreover, upon a relatively rare event of cell division within the healthy brain parenchyma, two sister cells show clear repulsion behavior, actively moving apart during the first three days after birth and reaching ~40 µm soma-to-soma distance by day 4 [23,25].

First seminal in vivo studies in anesthetized mice have shown that the fine microglial processes are extremely motile, continuously moving to interact with other cell types (neurons, astrocytes) or blood vessels [26,27]. This apparently random process movement enables them to monitor the current state of their immediate environment; a function which is known as surveillance. Surveillance is also important for the microglial contribution to proper wiring of the neuronal network during development [28] and the refinement of synaptic connectivity in the adult brain [29,30,31]. Therefore, microglia are thought to have an important role in the maintenance of the proper function and integrity of the neuronal circuitry. Mechanistically, the surveillance function of microglia depends on their membrane potential, which is under control of a K^+^-channel THIK-1 (TWIK-related halothane-inhibited K^+^-channel), which is tonically active under basal conditions [32].

The frequency of contacts between microglial processes and neurons is dependent on neuronal activity. Manipulations leading to reduced activity of neurons (e.g., sensory deprivation, reduction in body temperature, or application of the voltage-gated Na^+^-channel blocker tetrodotoxin) reduced the contact frequency between microglial processes and synapses [29]. Microglial processes can be recruited upon stimulation of glutamatergic receptors. During application of exogenous glutamate or NMDA (N-Methyl-D-aspartate), microglial processes extend towards neurons; and this recruitment is dependent on activation of neuronal NMDA receptors and accompanying Ca^2+^ influx, followed by ATP release from neurons and purinergic P2Y12 receptor-mediated stimulation on microglia [22,33]. Interestingly, a recent in vivo study has shown that a hyper- as well as a hypoactive state of the neural network can increase process outgrowth [34], indicating that any abnormal neuronal activity, irrespective of the direction of this abnormality, increases microglial process dynamics. Consistently, the dynamics of microglial surveillance is also changed by different kinds of anesthesia, known to modify neuronal activity [35,36]. During wakefulness, noradrenergic tone and activation of microglial β2 adrenoreceptors reduce the ramification and the surveillance territory of microglia as well as the surveillance speed of their processes [35,36]. The increased surveillance and more pronounced ramified morphology in the anesthetized brain seems to be a consequence of suppressed neuronal activity [35]. Importantly, however, also under awake conditions increases and decreases in baseline neuronal activity (i.e., shifts in the homeostatic set point) cause an increase in microglial arborization and process surveillance [35].

**Figure 1:**
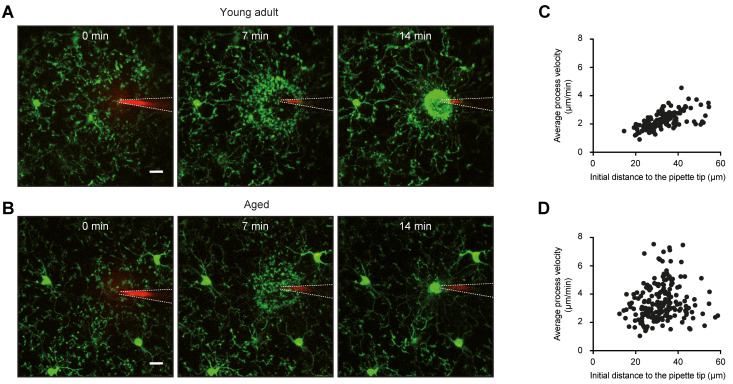
Microglial process extension towards an ATP source is less coordinated in the aged brain. (**A**,**B**) Maximum intensity projection images (80–100 μm below the cortical surface, 2 μm step size), obtained in vivo in the cortex of young adult (**A**) and aged (**B**) CX_3_CR1^GFP/+^ mice. Images show the extension of processes of GFP-positive microglia (green) towards a pipette containing 5 mM ATP and 200 µM of red fluorescent dye Alexa594 at three different time points (see time stamp) after ATP application. White dashed lines accentuate the locations of the pipette. (**C**,**D**) Scatter plots showing the relationship between the initial distance of the microglial process to the tip of the ATP-containing pipette and its average movement velocity in young adult (**C**) and aged (**D**) mice. Panels (**C**,**D**) are reproduced from ref. [39].

The multidirectional basal movement during resting conditions is rapidly changed towards directed movement upon injury or damage [26,37,38]. By doing so, microglial processes build up a spherical containment around the area affected by the insult, aiming at the removal of danger signals and cell debris and protection of the surrounding tissue. The built-up of a spherical containment (Figure 1A) requires a high degree of coordination between the individual processes of different microglial cells. Indeed, it turned out that the movement speed of the individual process is directly proportional to its initial distance from the DAMP-containing (insult) area [39,40] (Figure 1C). In this way, processes located further away move faster and quickly join up with the processes located closer to the insult area. Together, these processes are forming a symmetric and tight insulation compartment. In contrast to the mechanisms of basal process motility, THIK-1 is not important for directed process movement towards injury [32]. Instead, it relies on the activation of microglial P2Y12 receptors, mediated by ATP release from damaged cells [26,41]. Similar to the basal process movement, the microglial response towards (laser-induced) injury depends on the arousal state of the animal and is larger in the anesthetized mice [35,36]. In addition, the velocity of process movement towards laser-induced damage is higher under isoflurane anesthesia, as compared to the awake condition [42].

In summary, microglia in the young adult brain have a unique morphology characterized by small somata and long, elaborate processes, which are dynamic structures continuously surveying their environment in an apparently random manner. Upon damage or injury, processes switch to the directed movement towards the insult, relying upon a high degree of coordination between individual processes of different cells.

### 2.2. Ca^2+^ Signaling

Intracellular Ca^2+^ signaling is actively involved into both sensor and effector functions of microglia [34,43,44,45]. Due to technical reasons, however, it has long been challenging to study microglial Ca^2+^ signaling in vivo. Labeling individual microglial cells by means of single-cell electroporation, our group was the first to study Ca^2+^ signals in microglia residing in the intact brain [37]. The results of this study showed that under healthy homeostatic conditions, somatic Ca^2+^ signaling in microglia is infrequent, in contrast to that of other CNS cells, like neurons and astrocytes. However, microglia vividly respond with large Ca^2+^ transients to local neuronal damage in their immediate vicinity. These damage-induced Ca^2+^ transients were mediated by activation of metabotropic purinergic receptors, which are highly expressed in microglia [46], as well as Ca^2+^ release from the intracellular Ca^2+^ stores [37]. Subsequent studies substantiated our hypothesis that abundant somatic Ca^2+^ transients in microglia reflect pathological alterations in tissue homeostasis, e.g., aging [39,40], laser-induced focal brain injury [47], amyloid pathology [48], or peripheral inflammation [44,47]. Interestingly, the majority of Ca^2+^ transients evoked by laser-induced brain injury occurred in the microglial processes only [47]. This finding raised the suspicion that microglial Ca^2+^ signals might exist in several flavors: (i) The ones involving the entire cell (see Figure 1D in ref. [37]), as well as (ii) those restricted to subcellular microdomains, similar to what is known for astrocytes [49]. The use of a ratiometric genetically-encoded Ca^2+^ indicator Twitch-2B in cortical microglia [50] allowed monitoring not only transient, but also sustained alterations of the intracellular free Ca^2+^ concentration ([Ca^2+^]_i_). The results showed that in young adult mice under homeostatic conditions in vivo basal [Ca^2+^]_i_ in microglia is low, but increases significantly after laser-induced tissue damage, acute tissue slicing, or cell culturing [50], thus underscoring the ability of microglia to respond to the disturbance of tissue homeostasis not only with transient, but also with sustained changes in [Ca^2+^]_i_.

Change in the activity state of the surrounding neural network represents one of such disturbances of tissue homeostasis. Consistently, both an increase (caused, for example, by kainate-induced status epilepticus or activation of excitatory Designer Receptors Exclusively Activated by Designer Drugs (DREADDs)) and a decrease (induced by isoflurane anesthesia or inhibitory DREADDs) in neuronal activity increased intracellular Ca^2+^ signaling in microglia [34]. The latter Ca^2+^ signals also compartmentalized to microglial processes, similar to the laser damage-induced Ca^2+^ signals described above [47]. Increased incidence of Ca^2+^ transients in microglial processes was directly associated with process extension and outgrowth of new processes. Of note, both somatic and process-restricted Ca^2+^ signaling in microglia increased after kainate-induced status epilepticus, indicating that stronger stimuli are needed to activate somatic Ca^2+^ signaling in microglia.

In summary, the in vivo data obtained so far show that in the young adult brain the incidence of both somatic and process-restricted microglial Ca^2+^ signals under homeostatic conditions is low. However, it readily increases even in response to relatively mild disturbance of tissue homeostasis, as, for example, anesthesia or activation of excitatory/inhibitory DREADDs in the surrounding neurons, with process-restricted Ca^2+^ signals having lower and somatic Ca^2+^ signals having higher activation thresholds.

### 2.3. Gender-Specific Differences

The incidence and disease progression of psychiatric and neurological disorders with an inflammatory component differs between male and female individuals and the X chromosome is known for its high concentration of immune-related genes [51], pointing to the possible sex-specific operation of the brain’s immune system. Consistently, microglia express receptors for sex hormones and are, therefore, differently modulated by hormone status in males and females [51]. Moreover, microglial density was shown to differ in adult female and male mice. However, the literature results are not entirely consistent. Whereas in one study, microglial cell numbers were higher in the hippocampus of 3–24 months old female mice [52], a more recent study suggests that the density of microglia in young adult mice is higher in the hippocampus and cortex of male compared to female mice [53]. In yet another study, microglial density was similar in males and females in the somatosensory cortex of 2 months old mice [54].

Male and female microglia seem to react differently to brain damage, depending on the type of insult. Upon stab wound injury in the cortex/corpus callosum, a higher microglial cell density around the injury site was found in males. Of note, these microglia had a non-reactive and neuroprotective phenotype, consistent with a less severe stab wound-induced reduction in neuronal density in male compared to female mice [55]. In contrast, during ischemia, the damaged area was larger in males compared to females, and female microglia implanted into the brains of males had a protective effect [19]. Therefore, it seems that microglia react to the disturbance or damage of brain parenchyma in a sex- and injury-specific manner.

Adult female individuals have a higher susceptibility to autoimmune and inflammatory diseases [56], and a sex-specific comparison of transcriptomes of mouse microglia revealed that female microglia have a higher expression of genes, associated with inflammatory and immune responses (Figure S2 in ref. [54]). This points to a more immune-activated state of female microglia. At the same time, a detailed characterization of sex-specific properties of cortical microglia [53] revealed larger soma size and a higher expression of MHC proteins as well as some types of purinergic receptors in male microglia. In addition, higher baseline inward and outward conductances of the cell membrane and a stronger response to ATP were observed in male compared to female microglia [53]. Consistently, the functional in vivo studies from our group also document higher alertness of microglia in young adult male compared to female mice. Thus, under basal conditions the fraction of microglia showing spontaneous Ca^2+^ transients was significantly higher in male compared to female mice [40]. To substantiate the differences between young adult male and female mice, we analyzed a previously published RNAseq data set [57]. In this data set, the genes associated with inflammation were significantly upregulated in young male compared with female mice. The Ca^2+^ signaling-related pathways in general, as well as biological processes summarized under gene ontology annotations “release of sequestered calcium into cytosol,” “calcium-mediated signaling using intracellular calcium source,” or “positive regulation of cytosolic calcium ion concentration” were all upregulated in young adult male compared to female mice, but the difference observed did not reach the level of statistical significance [40]. These data are in good agreement with a study showing a higher transcriptional activation of NF-kB, a factor playing a key role in regulating the immune response, in male microglia [19]. When comparing transcription profiles of young adult male and female microglia, this study revealed that 79% of genes which were involved in NF-kB-mediated immune or inflammatory responses were more expressed in male mice [19]. Together, the literature data reveal a clear sex-specific signature of young adult microglia. Although the literature data are not entirely consistent, male microglia seem to be slightly more activated under homeostatic conditions. Microglia’s reactions to insults, however, might turn out to be damage- and brain region-specific.

## 3. Unexpected Alertness of Middle-Age Microglia

Epidemiological data on neurodegenerative diseases suggest that middle age is a critical period for disease onset and progression, as well as for the identification of promising treatment options able to stop or reverse pathology [58,59]. Interestingly, a recent study showed that middle age (e.g., ~12 months of age in mice) is also characterized by high intensity of oxidative stress and first aging-related changes in the brain’s energy metabolism [60]. Consistently, accumulating evidence suggests that the first aging-related switch in microglial function occurs with 9–12 months of age when animals are not even considered old. Microglia in middle-aged mice exhibited slight, not yet significant, alterations in morphology (e.g., an increase in soma volume and a reduction in process length), when compared to their counterparts in young mice [61]. There was, however, a significant reduction in the basal motility of microglial processes [61], suggesting that surveillance function might be impaired already at this early time point. Moreover, the brains of middle-aged mice contained a special subtype of microglia, which has also been described under several pathological conditions [62]. These so-called dark microglia were identified using electron microscopy-aided ultrastructural analyses and present with signs of oxidative stress, extensive contacts to synaptic elements, as well as high phagocytic activity, thus likely being involved in synaptic remodeling [62]. Because of the lack of in vivo methodology for the identification of dark microglia, a detailed functional characterization of these cells is still lacking.

Our in vivo data revealed a pronounced enhancement of spontaneous Ca^2+^ signaling in middle-age microglia in terms of frequency, amplitude, duration, and integral of individual Ca^2+^ transients [39,40], suggesting an activated, alerted functional state of middle-age microglia. This hyperactivity of Ca^2+^ signaling in individual cells together with an aging-related increase in the fraction of spontaneously active microglia represents a functional hallmark of the middle-aged brain. Although we did not evoke the mentioned above Ca^2+^ transients experimentally and therefore have to name them “spontaneous”, they are probably caused by damage/danger signals, accumulating in the brain parenchyma with age. Such microdamages might include the above mentioned accumulation of reactive oxygen species, ischemic events, death of individual cells, or rupture of small blood vessels.

As non-excitable cells, microglia rely on intracellular Ca^2+^ signaling to trigger their executive functions, e.g., increased processing and release of pro-inflammatory cytokines like IL-1β, IL-18 [63], TNF-α [64,65], or nitric oxide [66]. The latter might signal in an auto-/paracrine manner to sensitize microglia [34,44,45], which, in turn, might react much more vividly in case of subsequent pathology. Indeed, although basal levels of typical pro-inflammatory cytokines (TNF-α, IL-6, IL-1β) were similar in microglia from young and middle-aged mice, peripheral LPS challenge led to higher expression levels of these cytokines in brains of middle-aged mice [67]. The exact mechanisms, underlying the heightened pro-inflammatory response of middle-age microglia still have to be resolved but could, for example, be related to the differential expression of P2 receptors, known to vary with age and gender [68]. Some types of these receptors (e.g., P2X1, P2X3) were significantly higher expressed at middle-age, compared to young mice [68]. Interestingly, microglia in brains from middle-age mice showed a reduced proliferative capacity compared to their young counterparts, indicating that not all effector functions are affected in a similar way [67]. Interfering with the aging process at middle-age, e.g., by offering mice an enriched environment for the following 7.5 months, changed the microglial phenotype in aged mice [69]. Housing mice in an enriched environment from middle age onwards prevented aging-related deramification of microglial processes, downregulated the expression of inflammatory genes in the aged brain and improved behavioral outcome [70].

Together, the above described data suggest that the first switch in the functional signature of microglia occurs already at middle-age and is characterized by a reduced surveillance function, but heightened Ca^2+^ signaling, as well as heightened response to pro-inflammatory stimuli. Thus, the middle-age microglia are found in the overreacting, alerted state. Interfering with microglial over-reactivity at this early time-point might help to slow down the development of the aging-related pro-inflammatory brain state.

## 4. Functional Signatures of Aged Microglia

As already mentioned above, advanced age is characterized by a chronic low-grade inflammatory state of the brain, increased expression levels of pro-inflammatory cytokines, MHCII and complement receptors, as well as downregulation of anti-inflammatory genes [2,71]. Displaced self-molecules and reactive oxygen/nitrogen species, accumulating in the brain upon aging, inevitably impact functional properties of aged microglia. Typically, already under basal conditions, aged microglia produce more cytokines [72]. When facing a proinflammatory stimulus, aged microglial cells are hyperresponsive and produce larger amounts of cytokines over a longer timeframe [72,73]. This fact is likely responsible for prolonged sickness behavior in the elderly upon infection or illness, which might also be accompanied by depression and cognitive impairment [74], and fuels the development of age-related neurodegenerative diseases.

### 4.1. Transcriptional Signatures of Aged Microglia

Microglia obtained from aged (24-month-old) mice exhibit a different gene expression/transcriptional signature compared to their young counterparts [17,75,76]. The differentially regulated gene sets in the aged mouse brain were related to oxidative phosphorylation, mitochondria, lysosome- and phagosome-signaling pathways, as well as antigen presentation. Similar gene sets were differentially regulated in mouse models of neurodegenerative diseases [75]. This contrasted with the transcriptional profile of acutely activated microglia, which showed extensive NF-kB signaling, not found in aged microglia [75]. These findings suggest that the functional state of aged or chronically activated microglia differs from the one induced by an acute inflammatory insult. In addition, aged mouse microglia downregulated genes associated with the homeostatic function, like Tmem119 or P2ry12 [75]. Activation of NLRP3 inflammasome is another key mediator of the pro-inflammatory state of aged microglia, as NLRP3 depletion reduced aging-induced morphological activation of microglia, inflammation-induced increase in the brain expression of pro-inflammatory cytokines, as well as the age-related decline in memory and cognition [77,78].

The downregulation of genes associated with the homeostatic function of microglia, including the purinergic receptors P2ry12/P2ry13, Cx3cr1, and Tmem119, is also typical for so-called DAM (i.e., disease-associated microglia) [79,80]. These cells are further characterized by upregulation of genes involved in lysosomal, phagocytic, and lipid metabolism pathways, including several known AD risk factors, such as Apoe, Ctsd, Lpl, Tyrobp, and Trem2. DAM microglia are found in mouse models of neurodegenerative diseases (e.g., AD, Amyotrophic lateral sclerosis, and mouse models of tauopathy) as well as in postmortem brains of AD patients. In AD mice, for example, they are located in the vicinity of amyloid plaques and express high levels of transcripts engaged in phagocytic and lipid metabolism pathways. A fraction of DAM among microglia is also increasing with aging, amounting to 3% of all microglial cells in 20-month-old mice [79]. DAM can be considered as “fighting” microglia, whose primary function is to contain and remove the damage [80]. Induction of the DAM phenotype is triggered by danger molecules present on apoptotic bodies of dying neural cells, myelin debris, lipid degradation products, extracellular protein aggregates, as well as extracellular purines. This process depends upon TREM2 signaling and seems to be shared across various neurodegenerative diseases.

Microglial neurodegenerative phenotype (MGnD) [81], is also characterized by the suppression of homeostatic genes (e.g., P2ry12, Tmem119, Olfml3, Csf1r, Rhob, Cx3cr1, Tgfb1, Mef2a, Mafb, Sall1) and upregulation of inflammatory molecules (e.g., Apoe, Spp1, Itgax, Axl, Lilrb4, Clec7a, Csf1), being in this respect similar to DAM. This genetic makeup promotes the induction of pathways triggering phagocytosis, chemotaxis/migration, and cytokine release, thus allowing MGnD microglia to respond to neuronal injury in a defensive manner. The switch from homeostatic microglia to MGnD is regulated via the TREM2-APOE pathway [81]. Although functionally similar, DAM and MGnD phenotypes differ in their fine-grained transcriptional signatures [82].

Probably as a byproduct of their lifelong function as macrophages, microglia in the aged human, as well as rodent brain, accumulate un-degradable large inclusions, so-called lipofuscin granules consisting of unsaturated fatty acids, proteins, carbohydrates, metal ions as well as myelin fragments [5,83,84]. Approximately 50% of microglia in the aging brain have been shown to accumulate lipid droplets, mostly containing phospho- and glycerolipids, in their cytoplasm [82]. Because lipid droplet-laden microglia showed a specific transcriptome signature, this subset of microglia was designated by the authors LDAM (lipid-droplet-accumulating microglia). LDAM were shown to produce high levels of reactive oxygen and nitrogen species, release higher amounts of cytokines (e.g., IL-6, CCL3 or CXCL10) under control conditions, and to be defective in phagocytosis [82]. Thus, this phenotype can be regarded as a dysfunctional microglia.

In fact, impaired phagocytosis turned out to be a hallmark of aged microglia. Mechanistically, a recent paper identified the upregulation of CD22 in aged microglia as a negative regulator of phagocytosis [85]. CD22 is canonically expressed on B cells, where it negatively regulates B cell receptor signaling. Importantly, blocking CD22 promoted clearance of myelin debris or misfolded proteins like amyloid β oligomers and α-synuclein fibrils, reversed upregulation of inflammatory factors in microglia, and improved cognitive function of aged mice [85], suggesting that the impairment of phagocytosis during aging has an impact on cognitive function and pointing to CD22 as a promising target for the treatment of cognitive dysfunction in the elderly.

The gene expression profile of microglia obtained from laboratory rodents, which are kept in a controlled pathogen-free environment, might differ from that of humans. However, in a comparative study [86], an extensive overlap was observed between human and three independent mouse microglial gene expression data sets. Still, there were several human microglial genes (e.g., CD58, an adhesion molecule with a central role in the clustering of mature dendritic cells and T lymphocyte activation; ERAP1 and 2, peptides editing enzymes responsible for the trimming of N-terminal residues of MHC class I molecules, etc.), which were much less abundant in mouse microglia [86]. Noteworthy, genes with reduced expression during aging included many actin cytoskeleton-associated genes, cell surface receptors belonging to microglial sensome (e.g., P2RY12, IL6R, and TLR10), cell adhesion molecules, and cell surface receptors. Genes with higher expression during aging included integrin modulators DOCK1 and DOCK5, receptors CXCR4, CD163, and IGF2R, growth factor VEGFA and transcription factor RUNX3 [86]. Purinergic receptor P2RY12, mediating microglial chemotaxis, was also downregulated in aged human microglia, further highlighting the alteration of microglial sensing and motility with aging. In general, however, a comparison of the gene expression profiles between aged human and mouse microglia revealed a rather small number of significantly overlapping genes, with 14 common genes increasing and 9 common genes decreasing their expression upon aging [86]. The possible reasons for this mismatch are manifold including, but not limited to, the differences in host microbiota, different lifespans of the two species, human donors suffering from various pathologies, etc.

### 4.2. Morphology and Surveillance

**Figure 2:**
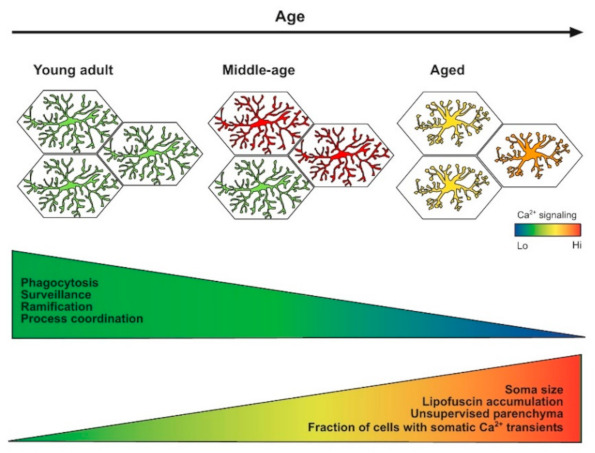
Schematic illustration of the functional states adopted by microglia along the axis of brain aging. See text for further explanations.

Although the number of microglial cells remains stable during the aging process both in humans and mice [23], microglia in the senescent brain adopt different morphological and functional phenotypes. In the aged human brain, microglial heterogeneity seems to be especially pronounced [84]. Here, a fraction of microglia shows a dystrophic morphology, characterized by the loss of fine ramifications, formation of cytoplasmic spheroids, beading, or fragmentations [87]. Importantly, it has to be noted that this extreme form of morphological microglial senescence can only be found in the human and not in the rodent brain, presumably because of differences in life span, environmental factors, and the degree of non-symptomatic brain pathology (e.g., microinfarcts) [84,88]. However, microglia in aged rodents also show specific morphological alterations indicative of a senescent phenotype (Figure 2), e.g., an increase in soma volume as well as reduced length, branching, and number of processes [61,78,89,90]. Due to this change in morphology, territorial domains of individual microglia are smaller, and the brain volume, covered by microglial processes, is reduced [78]. In addition, the expression of THIK-1, a K^+^ channel important for microglial surveillance (see above), and basal process motility are decreasing with age [11,61].

Surprisingly, although other groups have shown that directed movement of processes in the presence of a laser-induced injury is decelerated in the aged retina in situ [89] and the aged brain in vivo [61], data from our group suggested that the velocity of microglial processes, moving towards a point source of ATP, is increased in aged animals (Figure 1C, [39,40]). The reasons for these divergent observations are unclear, but may relate to the different experimental paradigms used in these studies. Laser-induced injury results in much more severe damage, compared to the insertion of an ATP-containing pipette, which more likely mimics small insults occurring in the brain under normal conditions. Importantly, we have also identified a severe impairment in the coordination of individual microglial processes from different cells, building up a spherical containment around the DAMP source (Figure 1D). Whereas in young adult mice, the initial distance of a process tip to the DAMP source is correlated with the mean velocity of extension (Figure 1B), this correlation decreases significantly in aged mice [39,40]. The final diameter of the containment formed by microglial processes, however, was similar in young adult, middle-aged, and old mice, suggesting that an equal number of processes contributed to the reaction in the different age groups [39,40].

In summary, microglia in the aged brain possess features of both a senescent, dystrophic (compromised surveillance function, uncoordinated process movement, reduced capability to phagocytose) as well as a reactive (accelerated velocity of process extension in response to minor tissue damage, longer and heightened production of inflammatory cytokines) phenotypes. Whether these functional phenotypes are represented by the described above subsets of microglia with different genetic signatures remains to be determined in the future.

### 4.3. Ca^2+^ Signaling

Brain aging is associated with an increase in the fraction of cells showing “spontaneous” microglial Ca^2+^ transients in vivo. As discussed above, the incidence of microglial cells with somatic Ca^2+^ transients is rather low in the young adult brain. It increases already in middle-aged animals and continues to stay high in aged mice [39,40].

Despite the high fraction of hyperactive cells, it is important to note that in comparison to middle-aged mice, spontaneous microglial Ca^2+^ transients in aged mice have a lower frequency, amplitude, duration, and AUCs, suggestive of impairment of intracellular Ca^2+^ signaling in individual cells. In addition, when challenged with a P2Y6 agonist UDP, microglia in the aged brain respond with significantly smaller Ca^2+^ transients compared to middle-aged microglia [39,40]. As activation of this receptor is involved in phagocytosis [91], these data suggest that phagocytic capabilities of microglia in the aged brain are impaired. These data provide further support for the accumulating body of evidence documenting the loss of phagocytic capacity of microglia upon aging [85,92,93,94]. Together with the impaired response of microglial processes to DAMPs or injury (see above), these data suggest that microglia in the aged brain are not able to effectively protect the brain parenchyma.

In summary, the Ca^2+^ signaling of microglia in the aged brain corresponds to a mixed phenotype combining features typical for the dysfunctional and the reactive states. Whereas the fraction of active cells is high, the strength of Ca^2+^ signals in individual cells decreases.

### 4.4. Gender-Specific Differences

Because microglia react to changes in their environment in a sex-specific manner (see above), it is conceivable to assume that brain aging is associated with a divergent microglia reaction in males and females. Indeed, a recent transcriptome study shows that the majority of age-related alterations in gene expression are sex-dependent [95]. Although the activation of inflammatory pathways with aging was evident in both females and males [95], the aging brain environment produced a stronger effect on gene transcription in female microglia, especially on the expression of genes involved in antigen presentation, innate immune response, or other inflammatory processes, as well as interferon response type I and the complement pathways [57,95], suggesting that female microglia age faster than their male counterparts. However, the genes, upregulated during the aging process, also included genes likely involved in tissue regeneration (e.g., Spp1, Gpnmb, and Dkk2), so that the overall functional outcome of the faster aging of microglial population in female mice remains to be determined.

Consistently, the functional in vivo data from our group also revealed sex-specific differences in aging-induced alterations of microglial Ca^2+^ signaling. Whereas in males the fraction of microglial cells showing spontaneous Ca^2+^ transients remained constant over the lifespan, this fraction gradually increased in female mice [40], amounting to almost 80% in aged female mice. The significant difference in microglial Ca^2+^ signaling between aged male and female mice [40] is again indicative of faster aging of female microglia. Together with fact that females are more prone to develop AD, these findings underline the necessity for in-depth study of sex-specific microglial reaction to aging, to elucidate underlying mechanisms of microglial dysfunction and establish new gender-specific treatment options.

## 5. Counteracting Aging of Microglia

### 5.1. Enhancing the α7 nAChR Function

An important role in microglia’s response to pro-inflammatory stimuli is played by nicotinic acetylcholine receptors of α7 subtype (α7 nAChRs). This nAChR subtype, represented by either a homopentamer or a heteropentamer including α7 and β2 subunits [96], is abundantly expressed within the brain. It is found in neurons, astrocytes, and microglia [97,98,99]. In contrast to nAChRs mediating fast synaptic transmission in neuromuscular junctions, the neuronal α7 nAChRs are located extrasynaptically and support the cell viability by transducing pro-survival and pro-proliferative signals [100,101]. The α7 nAChRs expressed in myeloid cells, including microglia, are involved in the cholinergic anti-inflammatory pathway. Upon activation, they attenuate the production of pro-inflammatory cytokines like IL-1β, IL-6, or TNFα [98,102,103,104,105]. Downregulation of α7 nAChRs in activated microglia results in reduced pro-survival and anti-oxidant signaling [106,107,108].

The aging-mediated increase in the level of oxidative stress is largely due to impaired mitochondrial function. The opening of the mitochondrial permeability transition pore (mPTP), resulting in membrane depolarization and uncoupling of the oxidative phosphorylation, plays an important role in neurotoxicity and is also involved in the aging process [109]. Navarro et al. showed that both primary glial cultures and microglia of animals treated with the α7 nAChR agonist PNU282987 showed a significant increase in mitochondrial mass and mitochondrial oxygen consumption [110]. Studies performed in our laboratory demonstrated that α7 nAChRs are expressed in the outer membrane of mitochondria and regulate the early events of mitochondria-driven apoptosis [111,112]. Activating α7 nAChRs attenuated cytochrome c release from isolated mitochondria, stimulated by apoptogenic doses of Ca^2+^ or H_2_O_2_ [113,114]. Neuroinflammation decreased the level of α7 nAChRs and stimulated the accumulation of Aβ_1-42_ in the brain mitochondria. This resulted in increased apoptogenic potency of Ca^2+^ [115], similarly to what was shown for mitochondria of aged animals [116].

Activating α7 nAChR with selective orthosteric agonist PNU282987 ameliorated spinal inflammatory infiltration and enhanced monocyte/microglia autophagy in the spinal cord from mice with experimental autoimmune encephalomyelitis (EAE), a model of multiple sclerosis [117]. PNU282987 significantly decreased the elevated expression of TNF-α, IL-1β, and calcitonin gene-related peptide, and likely also astrocytic/microglial proliferation in hippocampi of chronic migraine model rats [106]. Administration of another α7-nAChR agonist PHA-543613 reversed changes in pro-inflammatory and anti-inflammatory factors after chronic sleep deprivation [108].

A pro-inflammatory state of the aged brain promotes stroke [118], and several studies have shown that activating α7 nAChRs protects against ischemic stroke, inhibits the microglia-mediated inflammation [119], decreases lesion volume, behavior deficits, and increases the expression of antioxidant genes in microglia/macrophages [120]. Excessive inflammation in both the peripheral and central nervous systems may contribute to the initiation and maintenance of persistent neuropathic pain, which frequently affects older people [121,122]. The data described in ref. [123] suggest that α7 nAChRs on microglia represent a potential therapeutic target for treating neuropathic pain.

Neuroinflammation is a strong pathogenic factor for the development of age-related neurodegenerative disorders like Alzheimer’s and Parkinson’s diseases [124,125]. Acute neuroinflammation results in symptoms similar to early AD, like episodic memory loss and accumulation of Aβ peptides bound to α7 nAChRs. A similar effect could be achieved with the antibody against the extracellular domain of α7 nAChR subunit, demonstrating a critical role of this receptor in neuroinflammation [126]. In contrast to other nAChR subtypes, which are activated solely by acetylcholine, the α7 nAChRs can be also activated by choline [127]. Choline is constantly present within the cell, and its level was shown to influence the cell’s survival [128]. Lifelong choline supplementation significantly reduced amyloid plaque load and improved spatial memory in a transgenic mouse model of AD (APP/PS1 mice). These changes were linked to a decrease of the amyloidogenic processing of amyloid precursor protein, reductions in disease-associated microglial activation, and downregulation of the α7 nAChRs [129]. Other studies showed that acetylcholinesterase inhibitor galantamine (one of a few approved AD medications) sensitizes microglial α7 nAChRs to choline and induces Ca^2+^ influx into microglia, resulting in upregulation of Ca^2+^-dependent amyloid β phagocytosis [130]. Moreover, administration of α7 nAChR selective agonist 3-[(2,4-dimethoxy) benzylidene]-anabaseine dihydrochloride (DMXBA) attenuated brain Aβ burden and memory dysfunction in a mouse model of AD [131] as well as dopaminergic neurodegeneration and glial activation in a rat model of Parkinson disease [132]. Finally, even normal aging is accompanied by accumulation of brain Aβ_1-42_ [133], and activation of α7 nAChRs on microglial cells was neuroprotective in Aβ-induced neurotoxicity [134]. Therefore, selective α7 nAChR activation was suggested as a therapeutic approach to cure neuroinflammation-related and neurodegenerative disorders [135].

While the nAChRs are mostly known to the wider readership as classical ligand-gated ion channels, they can also function in a metabotropic way, by activating intracellular signaling pathways [136]. This type of signaling seems to be prevalent in non-excitable cells and is especially characteristic for α7 nAChRs, which desensitize quickly and therefore mostly signal in a metabotropic way. The α7 nAChRs were shown to bind heterotrimeric G proteins, to enhance Ca^2+^ release from intracellular Ca^2+^ stores, and to decrease the level of cAMP [137]. Consistent with this idea, several studies demonstrated that anti-inflammatory and neuroprotective effects in the brain can be achieved by using positive allosteric modulators (PAMs), “silent” agonists, or even antagonists, which influence the nAChRs sufficiently to trigger metabotropic signaling [138,139]. Application of type 2 PAMs seems attractive, because they might potentiate the effect of endogenous agonists like acetylcholine or choline [140]. Administration of α7 nAChR-specific type 2 PAM PNU120596 showed anxiolytic, pro-cognitive, and antidepressant-like effects. It also prevented activation of microglia and astrocytes and upregulation of proinflammatory cytokines in the hippocampus and prefrontal cortex of mice in response to peripheral inflammation [141]. In our studies, injecting mice with either orthosteric agonist PNU282987 or type 2 PAM PNU120596 prevented the inflammation-induced impairment of the mitochondrial function. However, in contrast to the stable and continuous pro-cognitive and anti-inflammatory effect of the agonist, the pro-cognitive effect of the type 2 PAM was transient and disappeared a week after the end of the injection cycle. Moreover, cessation of PNU120596 treatment resulted in a sharp increase of IL-1β and IL-6 levels in the blood of mice [142]. This result puts in doubt the idea of using type 2 PAMs instead of orthosteric α7 nAChR agonists for neuroprotection.

Taken together, these data demonstrate an important role of microglial α7 nAChRs for pro-survival and anti-oxidant signaling within the cholinergic anti-inflammatory pathway. This signaling supports the viability of the aging brain cells and the use of α7-selective drugs might pave the way for developing new therapies against age-related brain disorders.

### 5.2. Effect of Caloric Restriction

Dietary or caloric restriction (CR), i.e., the reduction of food intake without inducing malnutrition, has been shown to prolong life- and health-span in different organisms, from yeast to primates [143]. Furthermore, reduced food intake protects against cognitive decline and age-related diseases like AD and PD [144].

In non-human primates, CR reduced mortality and protected from age-induced pathology like diabetes, cancer, and cardiovascular disease, and prevented brain atrophy in subcortical regions [145]. In different mouse strains, CR delayed the onset of age-related pathology, e.g., the appearance of lymphoma, without necessarily prolonging the lifespan of the animals [146]. The beneficial effects of CR on lifespan and cognition are at least in part mediated by attenuation of oxidative stress, inflammation, and metabolic alterations associated with aging [60,144,147]. Indeed, CR decreased transcription of genes associated with inflammatory and stress response during the aging process in the cortex and cerebellum of mice [148]. Studies investigating different CR regimen in mice of different strains or genders show that some, but not all, of the CR-mediated effects depend on the sex, strain, and degree of restriction [60,146]. As an example, CR-mediated effects on hippocampal gene expression and improvement in learning and memory were larger in female mice [149].

Recent studies suggest that aging-mediated alterations in the microglial phenotype are also positively modulated by CR. In aged mice, a combination of low-fat diet and CR reduces the aging-induced activation of white matter microglia by preventing an aging-related increase in microglia number as well as upregulation of markers of microglial activation and phagocytosis (CD16/CD32, Mac-2, and Dectin-1) [150]. In the grey matter, CR prevented upregulation of the phagocytosis marker CD68 in some (e.g., corpus callosum), but not all, brain regions [151].

We studied the effect of CR in middle-aged and aged mice on the function of microglia under in vivo conditions [40]. We found that CR can shift the Ca^2+^ signaling properties back, closer to those seen in the respective younger age group. For example, the hyperresponsive in terms of Ca^2+^ signaling microglial phenotype, found in middle-aged mice, was reversed by 6 months of CR. Specifically, CR significantly decreased the frequency and duration of spontaneous Ca^2+^ transients in individual cells. After 6 months of CR, microglial Ca^2+^ signaling of calorie-restricted middle-aged mice resembled the phenotype observed in young control mice. After 12 months of CR, the hyporesponsive phenotype normally observed in aged mice was closer to the one found in control middle-aged mice. Noteworthy, even a short (6-week-long) CR starting at old age was able to rejuvenate some aspects of microglial Ca^2+^ signaling [40], thus documenting in addition to preventive also the therapeutic capabilities of CR. Neither CR protocol, however, prevented the aging-related increase in the fraction of the spontaneously active microglia. Interestingly, the ability of reduced food intake to alter microglial Ca^2+^ signaling was much more pronounced in male mice, indicating that CR has a sex-dependent effect on microglial function.

The rejuvenating effect of CR on microglia function was even more pronounced when considering the aging-related discoordination of microglial process movement towards a DAMP source (Figure 1). The faster and more disorganized movement of microglial processes towards an ATP source in the aged brain was reversed by both long- and even short-term CR [40]. These results demonstrate that CR is not only capable to prevent, but also to reverse the aging-related discoordination of microglial processes. As the coordinated movement of microglial processes is important for effective clearing/protection of the brain parenchyma, the CR-mediated improvement of this function is likely able to reduce the ongoing low-grade inflammation in the aged brain.

Taken together, CR has the potential to prevent or even restore the aging-induced impairment of microglial function, especially improving their neuroprotective capabilities. As CR is widely accessible and cost-effective, it represents a promising strategy to prevent inflammation and cognitive decline in the elderly.

### 5.3. The Role of Physical Exercise

Regarding their behavior, the CR mice were also much more mobile than their ad libitum fed counterparts. In fact, physical exercise itself is known to be anti-inflammatory and neuroprotective [152,153]. The cellular/molecular mechanisms involved include, but are not restricted to, (i) an improvement of hippocampal neurogenesis; (ii) increased expression levels of the brain-derived neurotrophic factor (BDNF) and insulin-like growth factor 1 (IGF-1); (iii) positive modification of the gut microbiota; (iv) reduction of the levels of pro-inflammatory (e.g., TNF-α and IL-1β) and increase in the levels of anti-inflammatory (e.g., IL-10) cytokines; (v) reduced expression of downstream targets of Toll-like receptor pathways, such as myeloid differentiation primary response gene 88 (MyD88) and NF-kB; (vi) upregulation of the OFF-signals, governing neuron-microglia interactions (e.g., CD200 and its receptor CD200R, soluble TREM2); (vii) secretion of anti-inflammatory adipokines and myokines, etc. [14,152,153,154]. Moreover, chronic endurance exercise enhances the production of metabolic factors, such as the NAD^+^-dependent protein deacetylase Sirtuin-1 (SIRT1) [154]. SIRT1 is known to inhibit the activity of NF-κB via deacetylation of the p65 subunit and to stimulate the antioxidant response via nuclear factor erythroid 2-related factor 2 (Nrf2)-mediated increase in the availability of antioxidant enzymes, including glutathione peroxidase and heme oxygenase-1 [154]. The increased synthesis of glutathione peroxidase, in turn, attenuates the release of pro-inflammatory factors TNF-α and IL-6 [154]. Another common mechanism bridging the physical exercise and CR is the liver-derived ketone body β-hydroxybutyrate, which is produced both during CR and during exercise, and has been shown to induce BDNF expression within the brain [155].

Voluntary running was able to counteract aging-mediated upregulation of microglial activation markers such as CD68, MHCII, and CD11b, as well as downregulation of neuronal genes related to synapses, axons, and neurotransmitters [156]. Interestingly, the exercise-mediated (i) reduction in the expression level of microglial inducible nitric oxide synthase, which promotes inflammation, and (ii) the enhancement of the mentioned above CD200-CD200R signaling, were also observed in a mouse model of PD [157]. Together, these data identify synergistic effects of physical exercise and CR, and call for more thorough analyses of common microglia-mediated cellular/molecular mechanisms in the future.

## 6. Conclusions

Recent studies revealed that functional states of microglia represent a multidimensional space. The states are rather quickly shifting along the activation axis in response to different pathologies, and slowly gliding between the three main age-related states during normal brain aging. The middle-age is the least studied among these age-related states. Besides the reduction in the basal motility of microglial processes, the data document the hyperreactivity of middle-aged microglia in respect to their Ca^2+^ signaling. It remains, however, to be determined whether this hyperreactivity is beneficial (e.g., helping to clear the parenchyma from accumulating byproducts of cell/tissue metabolism) or detrimental (e.g., enhancing the release of pro-inflammatory mediators, pruning vital synapses, or altering neuronal activity).

Much more is known about the aged microglia, which are represented by different transcriptional phenotypes. DAM/MGnD are considered fighting microglia, whose primary function is to contain and remove the damage. However, only 3% of microglia in the normally aged brain belong to this phenotype, whereas approximately 50% of cells belong to the dysfunctional LDAM microglia. Likely, these are the cells showing reduced Ca^2+^ signaling and basal process motility as well as impaired process coordination in vivo.

The future rejuvenation strategies might include pharmacological treatments, like the described above blockade of CD22 or activation of the NLRP3 inflammasome, choline supplementation or activation of α7 nAChRs. However, the possible side effects of such treatments must be thoroughly analyzed and taken into account. Alternatively, the modifications of the lifestyle, including, but not restricted to, physical exercise and caloric restriction, might be considered. These natural treatments have low side effects and positively impact the functional states of microglia. Although some of the underlying cellular/molecular mechanisms are still unclear, the data available to date identify (i) attenuation of oxidative stress as well as aging-related inflammation and metabolic changes, (ii) downregulation of microglial activation/phagocytosis markers, and (iii) increased expression levels of BDNF and IGF-1 as common mediators of positive effects caused by physical exercise and CR. As microglial Ca^2+^ signaling is tightly linked to their functional state, we anticipate that not only CR, but also physical exercise positively impact the Ca^2+^ homeostasis of microglia.

## Data Availability

All relevant data are included into the publication.
